# Temporal miRNA Biomarkers for Pupal Age Estimation in *Sarcophaga peregrina* (Diptera: Sarcophagidae)

**DOI:** 10.3390/insects16080754

**Published:** 2025-07-23

**Authors:** Yang Xia, Hai Wu, Sile Chen, Yuanxing Wang, Jiasheng Sun, Yi Li, Yadong Guo, Yanjie Shang

**Affiliations:** 1 Department of Forensic Science, XiangYa School of Basic Medical Sciences, Central South University, Changsha 410013, China; csu_xiayang@163.com (Y.X.); wuhai@csu.edu.cn (H.W.); 18216177266@163.com (S.C.); gdy82@126.com (Y.G.); 2 Public Security Forensic Center of Haidian, Beijing 100142, China; redstar790227@hotmail.com; 3 Changsha Public Security Bureau, Changsha 410013, China; hope_shadow@126.com (J.S.); 17673670205@163.com (Y.L.)

**Keywords:** *Sarcophaga peregrina*, microRNA (miRNA), pupal age estimation, differential expression analysis, forensic entomology

## Abstract

The pupal stage in necrophagous flies represents the longest and least morphologically distinct phase of development, posing a persistent challenge for accurately estimating postmortem intervals (PMI) in forensic investigations. Here, we present a novel molecular approach to pupal age estimation in *Sarcophaga peregrina*, a forensically important species, by profiling microRNA (miRNA) expression dynamics. High-throughput sequencing across early, mid, and late pupal stages identified 191 known miRNAs, of which nine exhibited distinct monotonic temporal trends. Six miRNAs (miR-210-3p, miR-285, miR-927-5p, miR-956-3p, miR-92b, and miR-275-5p) were validated by qRT-PCR and demonstrated consistent time-dependent expression patterns. Polynomial regression models revealed a strong correlation between miRNA abundance and developmental age (R^2^ = 0.88–0.99). Functional enrichment analyses of predicted miRNA targets highlighted their roles in key regulatory pathways, including ecdysteroid signaling, hypoxia response, autophagy, and energy metabolism. This study establishes, for the first time, a robust miRNA-based framework for estimating pupal age in forensic entomology, underscoring the potential of miRNAs as temporally precise biomarkers for PMI estimation.

## Simple Summary

Shortly after death, certain fly species rapidly colonize decomposing remains by laying eggs, initiating a biological timeline that can be used to estimate the minimum postmortem interval (PMI). Forensic scientists can use the development of these insects to estimate how long a person has been dead. However, during the pupal stage—the longest and least externally distinguishable part of a fly’s development—it is challenging to determine the developmental stage using external morphology. In this study, we explored a new way to solve this problem by examining small molecules called microRNAs (miRNAs), which naturally change as the fly develops. By tracking how specific miRNAs increase or decrease during pupal development, we created mathematical models to estimate the fly’s age with high accuracy. This new method could help forensic experts more precisely determine the time of death in criminal investigations.

## 1. Introduction

Forensic entomology is an interdisciplinary field integrating forensic science and entomology, focusing on the study of insect species colonizing decomposing remains. By examining the developmental stage, growth rate, and colonization patterns of necrophagous insects—alongside environmental variables such as temperature, humidity, and other ecological factors at the death scene—this discipline enables the construction of scientifically grounded models to estimate the minimum postmortem interval (PMI) with enhanced accuracy [1]. Extensive prior research utilizing entomological evidence from decomposing remains has revealed that the pupal stage in necrophagous flies often comprises more than half of the total developmental duration [2]. Consequently, accurate estimation of pupal age is critically important for improving the precision of postmortem interval (PMI) determination in forensic investigations [3].

However, during the mid-to-late pupal stages (i.e., approximately days 3 to 9 post-pupation under constant conditions), external morphological changes are often minimal or imperceptible, making it exceedingly difficult to accurately estimate pupal age based solely on outward appearance [2]. Traditional morphological approaches estimate pupal age by rearing specimens at constant temperatures until adult emergence. However, this method is time-consuming and susceptible to environmental and handling-related variables, often resulting in pupal mortality that compromises evidentiary value. More recently, internal anatomical examination has been employed to infer pupal age, offering faster results but relying heavily on subjective interpretation and specialized entomological expertise. These limitations hinder their routine application in forensic casework [4]. Thus, there is an urgent need to identify molecular biomarkers that change predictably with pupal development, enabling more efficient and objective age estimation.

With the advancement of molecular biology techniques in recent years, new avenues have emerged for accurately estimating the developmental age of necrophagous fly pupae. Studies have shown that differentially expressed genes (DEGs) in insects exhibit distinct temporal expression patterns during development, making them promising molecular markers for age estimation. Notably, Tarone et al. first applied gene expression data to estimate pupal age in *Lucilia sericata* (Meigen, 1826) (Diptera: Calliphoridae) [5]. Subsequently, Boehme, Shang, Hartmann, and others extended this approach to various forensically relevant fly species, further demonstrating the potential of molecular markers in forensic entomology [2,6,7].

Meanwhile, non-coding RNAs (ncRNAs) have garnered increasing attention in entomological research due to their pivotal roles in insect development, behavioral regulation, immune responses, and drug resistance [8]. In forensic entomology, ncRNAs are now being explored for their potential as molecular markers for pupal age estimation. Shang et al. conducted whole-genome sequencing of *Sarcophaga peregrina* (Robineau-Desvoidy, 1830) (Diptera: Sarcophagidae) and identified thousands of long non-coding RNAs (lncRNAs) that exhibited differential expression across pupal stages. Further analysis revealed that certain lncRNAs, such as SP_lnc5000, may regulate pupal development by modulating ecdysteroid signaling pathways and genes involved in cuticle formation; silencing these transcripts led to abnormal puparium development and failed eclosion [9]. Additionally, Wu et al. reported stage-specific expression patterns of circular RNAs (circRNAs) during the pupal phase of *Sarcophaga peregrina*, identifying four differentially expressed circRNAs and developing regression models based on their expression levels to estimate developmental age [10].

Among all classes of non-coding RNAs, microRNAs (miRNAs) are particularly well-suited for forensic applications due to their small molecular size, high chemical stability, and strong regulatory specificity, making them ideal candidates for expression profiling under complex postmortem conditions. miRNAs play essential roles in the spatial and temporal regulation of insect development and have been shown to exhibit stage-specific expression patterns. For instance, Hjelmen et al. performed high-throughput sequencing of miRNAs across the larval-to-pupal transition in the blow fly *Cochliomyia macellaria* (Fabricius, 1775) (Diptera: Calliphoridae), identifying 217 miRNAs, 17 of which were significantly differentially expressed during the pupal stage. Subsequent qPCR validation confirmed that miR-92b and bantam displayed consistent, monotonic changes over time, highlighting their potential as temporal biomarkers for pupal age estimation [11].

In summary, miRNAs represent a novel class of stable and sensitive molecular markers with significant potential for precise pupal age estimation in necrophagous flies. In this study, we employed *Sarcophaga peregrina*, a commonly encountered forensic species, as a model to systematically investigate the temporal dynamics of miRNA expression during pupal development. Based on a comprehensive literature review, this study is the first to profile miRNA expression during the pupal stage of Sarcophaga peregrina, providing novel insights into its molecular development. By identifying candidate miRNAs and exploring their putative regulatory roles through bioinformatic analyses, we aim to establish a reliable miRNA-based framework for molecular age estimation. This work provides both theoretical and experimental foundations for advancing the molecular toolkit of forensic entomology.

## 2. Materials and Methods

### 2.1. Collection and Synchronization of Necrophagous Fly Samples

Adult specimens of *Sarcophaga peregrina* were collected from a natural environment in Hunan Province, China, in July 2020 and used to establish a laboratory colony. Morphological identification was performed under the supervision of Prof. Lushi Chen (Guizhou Police Officer Vocational College), based on standard diagnostic characters, including male terminalia, as described in *Corpse-Feeding Flies in China* [12]. To confirm species identity, molecular identification was conducted on a randomly selected adult from the colony. DNA was extracted from dorsal thoracic muscle using a commercial genomic DNA extraction kit. The mitochondrial COI gene was amplified using primers barcode658-F (5′-GGTCAACAAATCATAAAGATATTGG-3′) and barcode658-R (5′-RAAACTTCAGGRTGACCAAAGAATCA-3′). PCR products were separated on a 1% agarose gel, purified, and subjected to Sanger sequencing. The resulting sequence was analyzed using the NCBI BLAST tool (https://blast.ncbi.nlm.nih.gov/Blast.cgi, accessed on 10 December 2020), confirming a high similarity to *Sarcophaga peregrina*. The colony was maintained under controlled environmental conditions: a constant temperature of 25 ± 0.5 °C, relative humidity of 75 ± 5%, and a 12:12 h light–dark photoperiod. To induce oviposition, approximately 20 g of fresh pig lung tissue was placed in a Petri dish to attract gravid females, allowing them to lay eggs that developed into first-instar larvae.

When approximately 80% of the larvae had undergone pupariation—defined by the onset of cuticle sclerotization and formation of the white puparium—300 individuals were collected at a single time point from a tightly synchronized cohort. Larval colonies were monitored at 4 h intervals under a stereomicroscope, and individuals were selected based on clearly defined morphological markers of pupariation, including anterior spiracle eversion and complete mouthpart retraction. All flies used were from a colony reared for over five generations under controlled laboratory conditions (25 ± 0.5 °C, 75 ± 5% RH, 12:12 h L:D). This ensured developmental consistency and minimized variation in downstream morphological and transcriptomic analyses [13,14]. These pupae were transferred into individual plastic containers lined with a 1 cm layer of sterilized wood shavings and incubated under the same controlled environmental conditions.

Starting from the white puparial stage, pupae were sampled at 24 h intervals over a 10-day period. At each time point, 15 pupae were randomly selected, immediately flash-frozen in liquid nitrogen, and stored at −80 °C for subsequent analysis. This process was independently repeated three times (i.e., three biological replicates), resulting in a total of 450 pupae (15 pupae × 10 time points × 3 replicates).

### 2.2. Observation and Documentation of Internal Pupal Morphology

After completion of time-series tissue sampling, 1 pupa per day was randomly selected from each biological replicate (n = 3) for internal morphological analysis, resulting in a total of 30 pupae (10 time points × 3 replicates). All selected individuals had been immediately flash-frozen in liquid nitrogen after collection and stored at −80 °C until analysis. Prior to dissection, pupae were slowly thawed at room temperature. The puparial case was carefully removed using ophthalmic scissors and insect pins, and internal features were examined under a ZEISS Stemi 508 stereomicroscope (Oberkochen, Germany). Internal morphological features—including head primordia, thoracic and abdominal segmentation, wing and leg bud development, eye pigmentation, and overall tissue organization—were examined and imaged. Morphological assessment focused on developmental features such as head primordia, segmentation, appendage differentiation, eye pigmentation, and organ maturation. Reference criteria for stage identification were adapted from our previous work [15], which established 10 intrapuparial sub-stages in *Sarcophaga peregrina* based on detailed histological analysis. These defined morphological benchmarks served as staging references to validate time point accuracy and developmental synchronization in the current study.

### 2.3. miRNA Extraction, Library Construction, and Sequencing

Based on well-documented morphological and molecular benchmarks in necrophagous fly development [16,17,18], we selected day 1, day 5, and day 9 to represent the early, middle, and late pupal stages, respectively. Day 1 corresponds to the onset of puparium formation and early organ primordia appearance; day 5 marks the phase of active tissue reorganization and differentiation; and day 9 coincides with late-stage pigmentation and near-complete adult morphogenesis.

For miRNA sequencing, 30 pupae were randomly selected per time point from the existing pool of samples already collected as described in Section 2.1. Specifically, from the previously collected 45 pupae (15 per replicate × 3 replicates) per day, 10 pupae per replicate (i.e., 30 total) were used for RNA extraction at each time point, resulting in 90 total pupae (3 stages × 30 pupae). No new individuals were sampled for sequencing; these specimens were subsampled from the larger cohort previously described. Total RNA was extracted using a small RNA-specific kit provided by Accurate Biotechnology Co., Ltd. (Changsha, China), following the manufacturer’s instructions. RNA concentration and purity were assessed using a NanoDrop 2000 spectrophotometer (Thermo Fisher Scientific, Waltham, MA, USA), and RNA integrity was evaluated with an Agilent 2100 Bioanalyzer (Santa Clara, CA, USA). Only samples with an RNA Integrity Number (RIN) ≥ 7 were selected for downstream library preparation. All biological replicates used in the study consistently met this threshold, ensuring uniform RNA quality across all time points. Library construction included sequential ligation of 3′ and 5′ adapters, reverse transcription, PCR amplification, and library purification to generate small RNA libraries. Libraries that passed quality control were subjected to high-throughput sequencing using the Illumina platform (OE Biotech Co., Ltd., Shanghai, China) with a single-end 50 bp read mode.

### 2.4. Data Preprocessing and miRNA Expression Analysis

Raw sequencing data were exported in FASTQ format. Adapter sequences were trimmed using Cutadapt (version 1.14), and reads shorter than 15 bp or longer than 41 bp were discarded. Sequence quality was evaluated using the FASTX-Toolkit (version 0.0.13), and only high-quality reads with Q20 scores ≥ 80% were retained. Reads containing ambiguous nucleotides (N) were removed using NGSQC Toolkit (version 2.3.3) to obtain clean reads for downstream analysis. Clean reads were aligned against the Rfam database to annotate and remove non-miRNA sequences such as rRNA, tRNA, snRNA, and snoRNA. Repeat sequences were further eliminated using RepeatMasker (version 2.0.2). Unannotated reads were subjected to novel miRNA prediction using miRDeep2, and their secondary structures were assessed with RNAfold. miRNA expression levels were normalized and reported as transcripts per million (TPM).

### 2.5. Differential Expression Analysis, Temporal Trend Identification, and Functional Enrichment

Differential expression analysis was conducted using the DESeq2 R package (v1.30), employing a standard negative binomial distribution model to perform pairwise comparisons of miRNA expression across the three key pupal developmental stages (P1, P3, and P5). Significantly differentially expressed miRNAs were identified using DESeq2 with a Benjamini–Hochberg adjusted q-value < 0.05 and an absolute log_2_ fold change (|log_2_FC|) > 1. These biostatistical thresholds ensured stringent control of false discovery rates and reliable identification of temporal miRNA expression changes. To further explore temporal expression dynamics, Short Time-series Expression Miner (STEM, v1.3.11) was employed for clustering analysis of differentially expressed miRNAs. TPM-normalized and log_2_-transformed expression values were used as input, with the maximum number of model profiles set to 16. Temporal expression clusters with a false discovery rate (FDR) < 0.05 were considered statistically significant and selected as candidate time-series miRNA profiles.

Target gene prediction for the differentially expressed miRNAs was performed using an integrative approach combining three algorithms: miRanda, TargetScan, and RNAhybrid. The input included significantly differentially expressed miRNAs and a custom *Sarcophaga peregrina* Unigene transcriptome database. High-confidence miRNA–mRNA interactions were defined by the following criteria: presence of 8mer or 7mer-m8 seed matches, binding free energy ≤ –25 kcal/mol, a prediction score ≥ 150, and concordant identification by at least two of the three algorithms.

Gene Ontology (GO) enrichment analysis was performed using the topGO R package (version 2.44.0), applying Fisher’s exact test to identify significantly enriched terms across three categories: biological process (BP), molecular function (MF), and cellular component (CC). A false discovery rate (FDR) threshold of <0.05 was used to determine statistical significance. Kyoto Encyclopedia of Genes and Genomes (KEGG) pathway enrichment analysis was conducted using the KOBAS 3.0 platform. Unigene sequences were aligned to the *Drosophila melanogaster* reference database via BLAST, and significantly enriched pathways were identified using a Benjamini–Hochberg adjusted *p*-value < 0.05. Enrichment results were visualized using bar charts and circular plots and used for downstream functional interpretation and pathway reconstruction (See Section 3.5). Representative miRNA–target gene regulatory networks were visualized using Cytoscape v3.9.0.

### 2.6. Selection of Candidate miRNAs and qRT-PCR Validation

Nine candidate miRNAs exhibiting representative differential expression trends were selected for validation by quantitative real-time PCR (qRT-PCR). Reverse transcription was performed using a 3′-end polyadenylation-based method with the miRNA 1st Strand cDNA Synthesis Kit (AG11716, Accurate Biotechnology, Changsha, China), and primers were designed using Primer 5.0. U6 small nuclear RNA was used as the internal reference gene (Table 1).

qRT-PCR was conducted on an ABI 7500 Real-Time PCR System using SYBR^®^ Green Premix Pro Taq HS qPCR Kit (ROX Plus, Accurate Biotechnology). Independent biological samples were used for each time point, with three technical replicates per sample. The average cycle threshold (Ct) values were used to calculate relative expression levels using the 2^–ΔΔCt^ method. miRNAs that showed consistent validation results were further quantified across all ten pupal time points to generate temporal expression profiles 2.1.

### 2.7. Data Analysis and Model Construction

Six miRNAs validated by qRT-PCR (miR-210-3p, miR-285, miR-927-5p, miR-956-3p, miR-92b, and miR-275-5p) were used as input variables to construct fourth-order polynomial regression models. These models were designed to capture the nonlinear relationship between miRNA expression levels and developmental time (in days). All modeling and visualization were performed using R. The goodness-of-fit for each model was evaluated using the coefficient of determination (R^2^) and the residual sum of squares (RSS).

## 3. Results

### 3.1. Stage-Specific Morphogenesis of Sarcophaga peregrina Pupae

**Figure 1:**
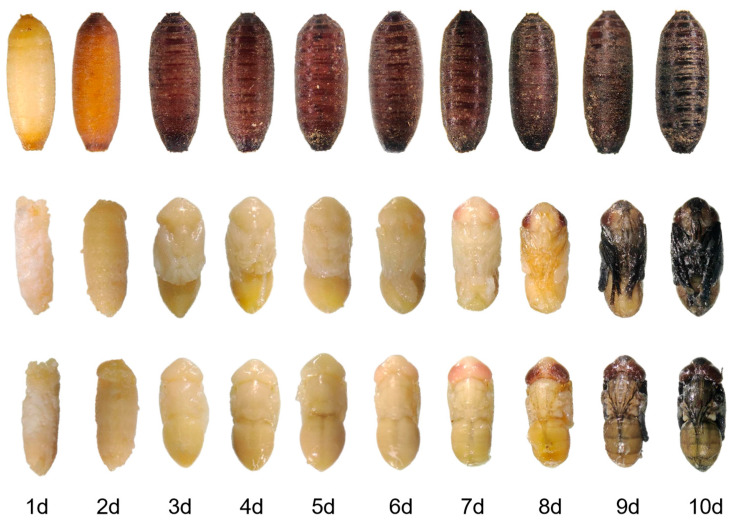
Morphological changes during pupal development of *Sarcophaga peregrina* from day 1 to day 10.

To characterize the temporal morphological transitions during pupal development in *Sarcophaga peregrina*, we conducted systematic observations of both external and internal structures under controlled conditions (25 ± 0.5 °C) over a 10-day period. Although the pupal exterior remained relatively unchanged and did not present distinct visual cues for developmental staging, internal tissue remodeling followed a highly time-dependent trajectory. Building on our previous detailed staging of intrapuparial development using morphological and histological analyses [15], we classified the pupae into ten developmental stages based on daily observations (Supplementary Figures S1–S10). Beginning on day 1, the pupal case gradually darkened from pale yellow to reddish brown, ultimately reaching a dark brown mature state by days 8–10. Internally, early signs of organogenesis were evident as early as day 2, with the emergence of head and limb primordia. Between days 3 and 5, the thorax and wing pads became increasingly defined, signaling active tissue reorganization. By day 7, eye pigmentation and wing development were prominent, and by days 8–9, most internal organs were differentiated with substantial pigment deposition. By day 10, the adult morphology was largely complete. These time-structured morphological changes provide a developmental framework for interpreting temporal shifts in molecular expression profiles (Figure 1).

### 3.2. Quality Assessment of Small RNA Sequencing Data

A total of nine small RNA libraries were generated from pupae sampled at three developmental time points (days 1, 5, and 9), with each library yielding over 40 million raw reads. After quality filtering, approximately 12–14 million clean reads per sample were retained, with >98% of bases achieving a Q20 quality score and minimal N content, indicating high sequencing fidelity. The number of unique clean reads ranged from 200,000 to 600,000, suggesting a certain degree of expression bias among miRNAs (Table S1). Read length distribution analysis revealed a prominent peak at 22 nucleotides across all samples, consistent with the expected size of mature eukaryotic miRNAs and confirming the efficiency of library construction and enzymatic digestion (Figure S11). The alignment rate of clean reads to the reference genome exceeded 94%, with uniquely mapped reads accounting for 40–55% (Table S2), indicating reliable mapping specificity. Approximately 0.7–0.8% of reads were annotated as known miRNAs in the miRBase database, suggesting the presence of potentially novel miRNA species within the dataset (Table S3). Notably, day 5 (P3) samples exhibited a markedly higher number of unique miRNAs compared to days 1 (P1) and 9 (P5), hinting at a more complex regulatory landscape during mid-pupal development (Table S4). Overall, the dataset demonstrated high quality, strong reproducibility, and broad expression coverage, providing a robust foundation for downstream differential expression analysis and novel miRNA discovery.

### 3.3. Temporal Patterns of miRNA Expression Across Pupal Development

**Figure 2:**
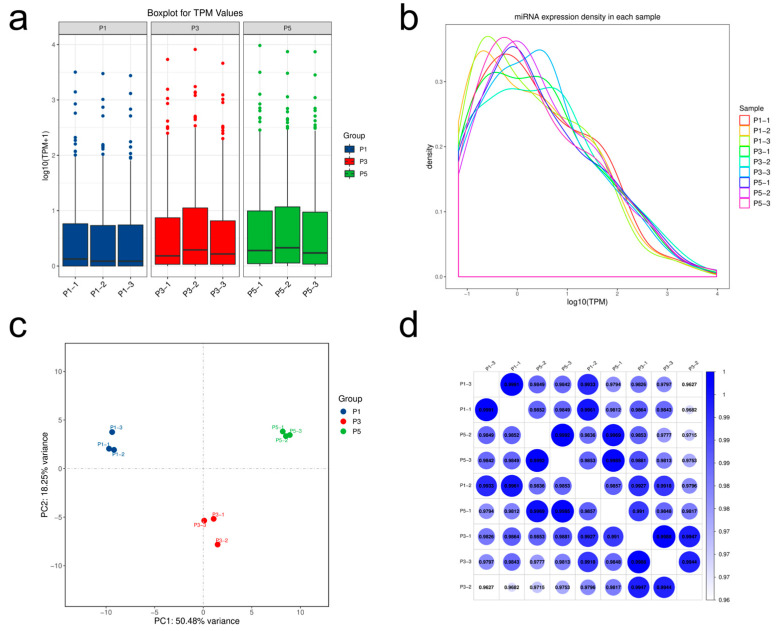
Comprehensive analysis of miRNA expression dynamics during pupal development in *Sarcophaga peregrina*. (**a**) Boxplot of log_10_(TPM + 1) shows increasing miRNA expression from P1 to P5, with individual dots representing sample-level expression values. (**b**) Density plots indicate a shift toward higher expression levels in P3 and P5. (**c**) PCA reveals clear stage-specific clustering, with PC1 explaining 50.48% of variance. (**d**) Pearson correlation heatmap shows high within-group similarity and intergroup divergence, with circle size and color intensity indicating the strength of correlation between samples.

To investigate dynamic changes in miRNA expression during the pupal development of *Sarcophaga peregrina*, expression levels were normalized using transcripts per million (TPM) and analyzed across three developmental stages (Table S5). Overall, miRNA abundance exhibited a progressive increase as development advanced. Boxplot analysis revealed that samples from the mid (P3) and late (P5) pupal stages had higher median log_10_(TPM + 1) values and a greater proportion of highly expressed miRNAs compared to early-stage samples (P1), suggesting broader regulatory activity in later stages (Figure 2a). Density plots further supported this observation, with P3 and P5 curves shifted toward higher expression values, while P1 showed enrichment in the low-abundance range (Figure 2b). Correlation heatmaps indicated strong consistency within each group (Pearson’s r > 0.99), but reduced correlation across different stages, reflecting stage-specific miRNA expression patterns (Figure 2c). Principal component analysis (PCA) corroborated these findings, with distinct clustering of P1, P3, and P5 samples along the first principal component (PC1), which accounted for over 50% of total expression variance. Collectively, these results demonstrate a temporally structured and robust miRNA expression landscape during pupation, supporting its potential for age estimation and functional inference.

### 3.4. Differential Expression Analysis of miRNAs Across Developmental Stages

**Figure 3:**
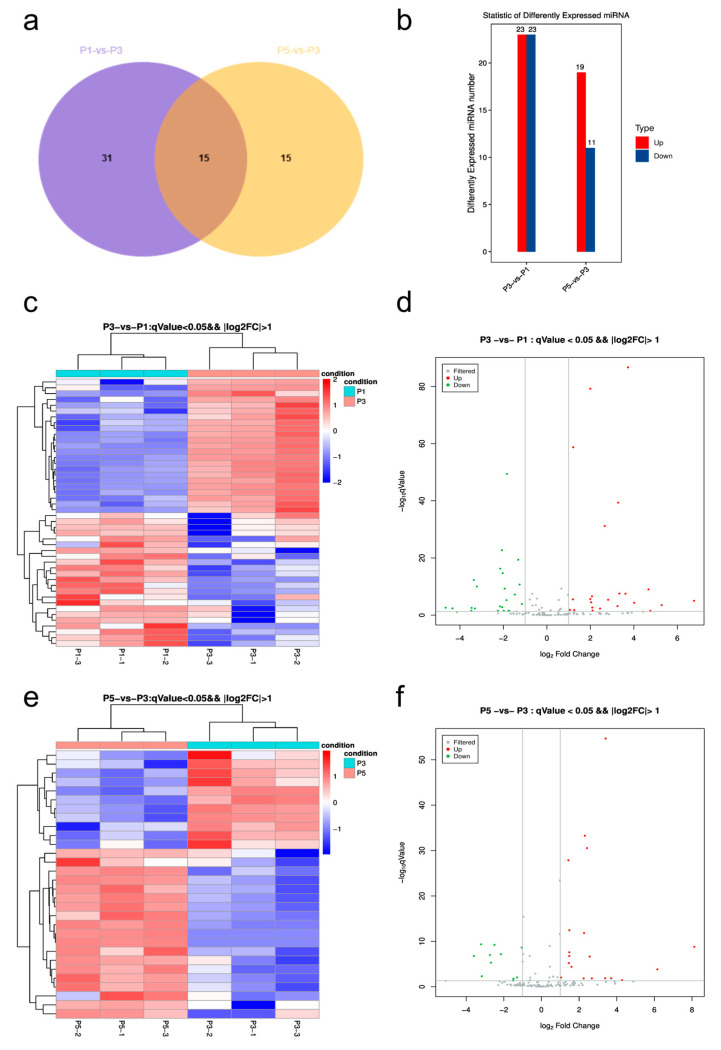
Differentially expressed miRNAs across pupal stages in *Sarcophaga peregrina*. (**a**) Venn diagram showing overlapping differentially expressed miRNAs between P3 vs. P1 and P5 vs. P3 comparisons. (**b**) Bar chart of upregulated and downregulated miRNA counts in each comparison. (**c**,**e**) Heatmaps of differentially expressed miRNAs in P3 vs. P1 and P5 vs. P3 groups, respectively. (**d**,**f**) Volcano plots displaying the magnitude and significance of expression changes. Some miRNAs show log_2_FC > 6–8, indicating strong stage-specific regulation. Gray vertical lines indicate the threshold of |log_2_FoldChange| = 1 used to define differential expression.

To capture dynamic shifts in miRNA expression during pupal development, differential expression analysis was performed for two pairwise comparisons: P3 vs. P1 and P5 vs. P3. miRNAs were considered significantly differentially expressed if q-value < 0.05 and |log_2_FC| > 1. A total of 46 miRNAs were identified in the P3 vs. P1 comparison (23 upregulated, 23 downregulated), and 30 miRNAs were detected in the P5 vs. P3 group (19 upregulated, 11 downregulated), with 15 miRNAs overlapping between comparisons (Figure 3a,b), indicating sustained expression regulation across stages. Hierarchical clustering revealed more pronounced divergence between P3 and P1, with P3 samples showing consistently elevated levels for multiple miRNAs (Figure 3c). The corresponding volcano plot demonstrated a balanced distribution of significantly altered miRNAs, with some upregulated miRNAs exhibiting log_2_FC values > 6 (Figure 3d). Although differences between P5 and P3 were more moderate overall, several miRNAs still showed persistent upregulation or downregulation at the late stage, including cases where log_2_FC exceeded 8 (Figure 3e,f), suggesting continued transcriptional regulation in late pupation.

Notably, several miRNAs exhibited consistent expression trends across both comparisons. miR-133, miR-285, miR-210-3p, miR-956-3p, and miR-957-3p were upregulated from early to late stages, while miR-92a and miR-92b showed progressive downregulation, suggesting distinct roles in stage-specific developmental regulation.

### 3.5. Target Prediction and Functional Enrichment of Differentially Expressed miRNAs

In the P3 vs. P1 comparison, 46 differentially expressed miRNAs (23 upregulated, 23 downregulated) were subjected to target prediction using three algorithms—miRanda, TargetScan, and RNAhybrid. High-confidence miRNA-mRNA interactions were retained based on stringent thresholds (total score ≥ 150, binding energy ≤ −25 kcal/mol, and presence of 8mer or 7mer-m8 seed sites), resulting in 96 predicted pairs involving 21 miRNAs and 75 target transcripts. The same strategy applied to the P5 vs. P3 comparison yielded 43 pairs involving 13 miRNAs and 43 target genes (Tables S6–S8). Representative examples include dme-miR-34-5p targeting the 5′UTR of evm.model.Contig102.10 with two 7mer-m8 sites (ΔG = −67.28 kcal/mol), and dme-miR-210-5p binding the 3′UTR of evm.model.Contig185.36 with high stability (ΔG = −30.80 kcal/mol), both suggesting strong post-transcriptional repression (Figure S12).

**Figure 4:**
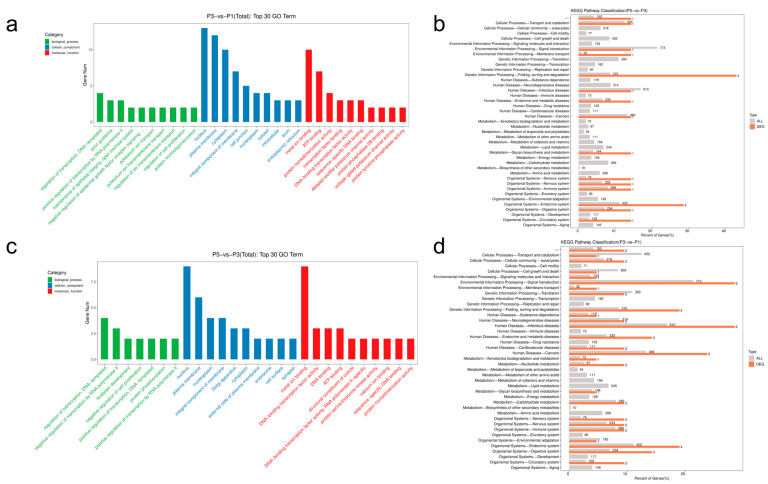
Functional enrichment of predicted target genes of differentially expressed miRNAs in *Sarcophaga peregrina* pupae. (**a**) Gene Ontology (GO) enrichment of P3 vs. P1 target genes, showing the top 30 significant terms across biological processes (BP), molecular functions (MF), and cellular components (CC). (**b**) KEGG pathway enrichment for P3 vs. P1 targets, highlighting involvement in MAPK, Wnt, Hippo, and ecdysone signaling pathways, as well as glycolysis/gluconeogenesis. (**c**) GO enrichment of P5 vs. P3 target genes, emphasizing roles in redox balance, hypoxia response, and autophagy. (**d**) KEGG pathway enrichment for P5 vs. P3 targets, with strong enrichment in HIF-1, FoxO, autophagy, and oxidative phosphorylation pathways.

GO enrichment analysis revealed distinct stage-specific regulatory roles (Figure 4a,c). In P3 vs. P1, targets were enriched in transcriptional regulation, chitin-based cuticle development, and ion transport, with dominant molecular functions including DNA-binding transcription factor activity and ATP binding—indicating active cuticle remodeling and metabolic activation during mid-pupation. In contrast, the P5 vs. P3 group showed enrichment in oxidation–reduction processes, hypoxia response, and autophagy, along with oxidoreductase activity and heme binding, pointing to metabolic suppression and stress adaptation in late pupae. KEGG pathway analysis supported the GO findings (Figure 4b,d). Early- to mid-pupal targets were significantly enriched in MAPK, Wnt, Hippo, and Ecdysone signaling pathways, alongside upregulated glycolysis/gluconeogenesis, highlighting coordinated cell proliferation, hormone regulation, and energy metabolism. By late pupation, HIF-1, FoxO, autophagy, and oxidative phosphorylation pathways became dominant, reflecting hypoxia-driven metabolic remodeling.

Collectively, these data support a biphasic regulatory model of miRNA-mediated control during pupal development in *Sarcophaga peregrina*. In early to mid-stages, upregulated miRNAs such as miR-34-5p and let-7-5p repress structural and endocrine regulators (e.g., cuticle proteins, *Kr-h1*), acting through MAPK/Wnt–Ecdysone signaling to initiate cuticle sclerotization and molting transitions. In later stages, hypoxia-responsive miRNAs like miR-210-5p target key metabolic genes (e.g., E75), activating HIF-1/FoxO–autophagy pathways to downregulate metabolism and enhance stress resilience. This temporal regulatory framework provides functional insights and candidate molecular markers for pupal age estimation.

### 3.6. Temporal Trend Analysis of Differentially Expressed miRNAs

To investigate the temporal expression dynamics of differentially expressed miRNAs across pupal stages (P1, P3, P5), we employed Short Time-series Expression Miner (STEM) clustering. Sixteen predefined temporal profiles were generated, among which only Profile 11 showed significant enrichment (*p* = 0.0017, FDR = 0.027). This cluster displayed a consistent upward trend (0.0→1.0→1.0) and included 18 miRNAs, most of which were progressively upregulated from early to late stages.

**Figure 5:**
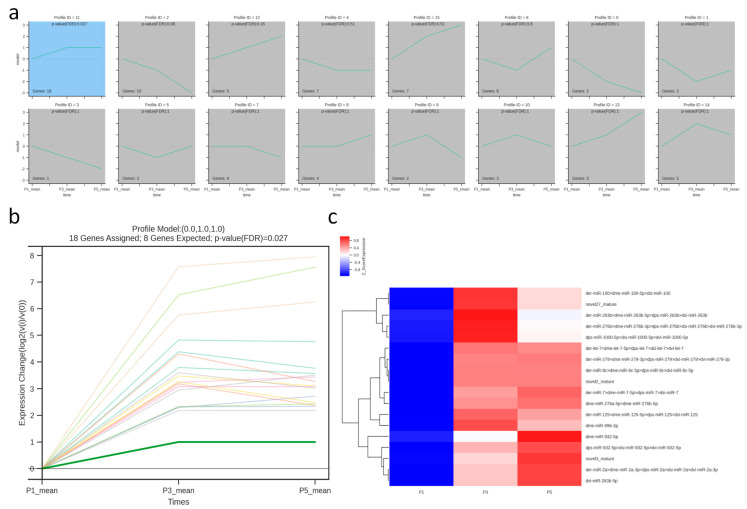
Time-series clustering of differentially expressed miRNAs in *Sarcophaga peregrina* pupae using STEM. (**a**) STEM clustering identified 16 temporal profiles; only Profile 11 showed significant enrichment (FDR = 0.027). (**b**) Line plot of Profile 11 showing a consistent upregulation trend from P1 to P5. (**c**) Heatmap of the 18 miRNAs assigned to Profile 11, most of which exhibit increasing abundance over time. Different colored lines represent individual miRNAs within Profile 11 and are used solely for visual distinction.

Notably, several miRNAs in Profile 11 were functionally consistent with the results of target gene enrichment. For instance, dme-let-7-5p, dme-miR-263b-5p, and dme-miR-276b-3p were associated with pathways such as ecdysone signaling, oxidative phosphorylation, and metabolic remodeling. These findings suggest that the time-dependent upregulation of Profile 11 miRNAs may play crucial roles in regulating pupal development and represent promising molecular markers for developmental stage estimation (Figure 5).

### 3.7. qRT-PCR Validation of Candidate miRNAs

To validate the expression trends of differentially expressed miRNAs identified via high-throughput sequencing, we performed qRT-PCR analysis on nine representative candidates with distinct temporal patterns. Validation was conducted at three key time points—day 1 (D1), day 5 (D5), and day 9 (D9)—and compared against transcriptomic TPM values. Most miRNAs showed expression trends consistent with the sequencing data, with a strong correlation observed between RQ (relative quantification) and TPM levels.

**Figure 6:**
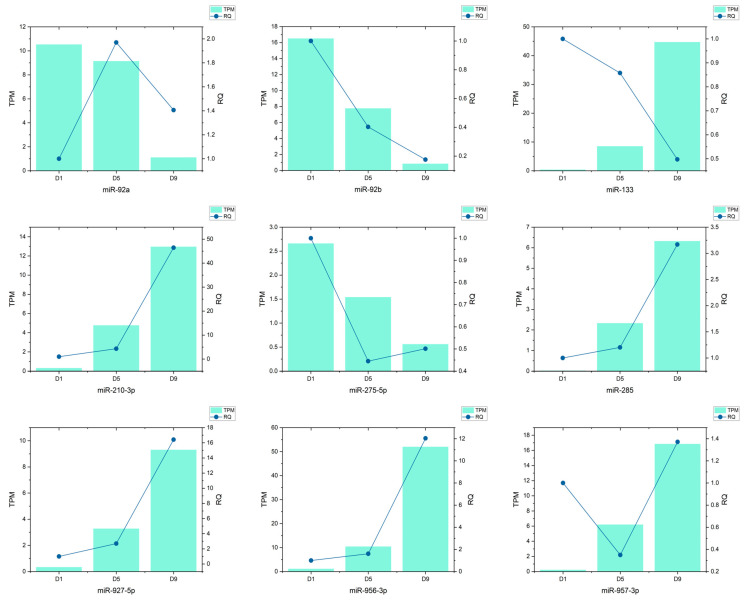
Comparison of expression levels of nine candidate miRNAs at different pupal stages (TPM and qRT-PCR).

Among them, six miRNAs-miR-285, miR-92b, miR-210-3p, miR-275-5p, miR-927-5p, and miR-956-3p-exhibited significant and monotonic changes across developmental stages. These were subsequently selected for extended qPCR profiling from day 1 to day 10. Their consistent expression dynamics across time points highlight their potential as temporal biomarkers, and they were further incorporated into regression and classification models for continuous pupal age estimation in *Sarcophaga peregrina* (Figure 6).

### 3.8. Dynamic Modeling of miRNA Expression Trends During Pupal Development

**Figure 7:**
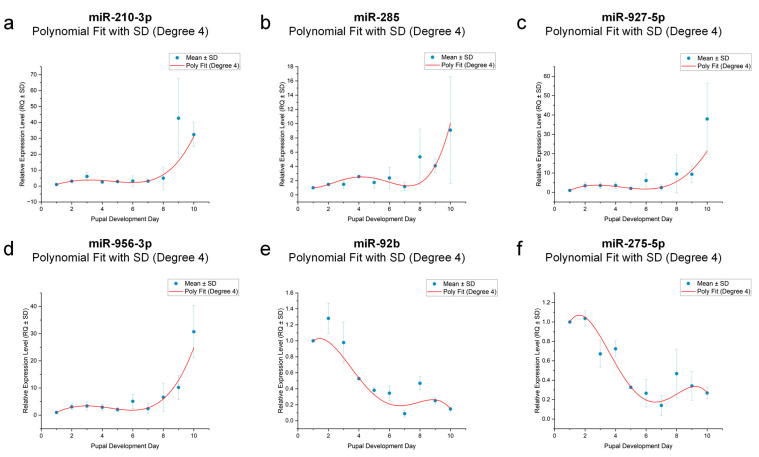
Polynomial regression modeling of miRNA expression across pupal development. (**a**) *miR-210-3p* expression trend fitted with a 4th-degree polynomial curve. (**b**) *miR-285* expression trend fitted with a 4th-degree polynomial curve. (**c**) *miR-927-5p* expression trend fitted with a 4th-degree polynomial curve. (**d**) *miR-956-3p* expression trend fitted with a 4th-degree polynomial curve. (**e**) *miR-92b* expression trend fitted with a 4th-degree polynomial curve. (**f**) *miR-275-5p* expression trend fitted with a 4th-degree polynomial curve. Each panel displays mean ± SD and the fitted curve (red) over the 10-day pupal stage.

To further define the temporal dynamics of candidate miRNAs and their functional relationship with pupal age, we performed fourth-order polynomial regression modeling using qRT-PCR data from six validated miRNAs: miR-210-3p, miR-285, miR-927-5p, miR-956-3p, miR-92b, and miR-275-5p. All six miRNAs exhibited clear nonlinear expression patterns across the 10-day pupal timeline (Figure 7).

Specifically, miR-210-3p, miR-285, miR-927-5p, and miR-956-3p displayed upward trajectories, peaking at days 9–10, suggesting their utility as molecular markers for mid-to-late pupal stages. In contrast, miR-92b and miR-275-5p showed decreasing trends during the early 4–6 days, followed by a plateau, making them more suitable for estimating early to mid-pupal age.

All regression models demonstrated strong fit, with coefficients of determination (R^2^) ranging from 0.879 to 0.992 (Table 2), indicating significant correlations between miRNA expression levels and pupal developmental age. Among them, miR-275-5p (R^2^ = 0.997), miR-92b (R^2^ = 0.992), and miR-285 (R^2^ = 0.974) showed particularly high model performance, reflecting clear time-dependent expression patterns under controlled conditions. These results support the feasibility of using miRNA expression levels as continuous molecular indicators for pupal age estimation in *Sarcophaga peregrina.*

## 4. Discussion

This study aimed to characterize the temporal expression patterns of microRNAs (miRNAs) during pupal development in the necrophagous fly *Sarcophaga peregrina*, a species of forensic importance. We performed high-throughput miRNA sequencing at early, middle, and late pupal stages, followed by differential expression and functional prediction analyses. Although miRNAs have been extensively studied in insects, investigations focusing specifically on necrophagous Diptera—particularly those relevant to forensic applications—remain limited, especially during the pupal stage. Previous molecular approaches to pupal age estimation in such species have primarily concentrated on mRNA expression profiles. In recent years, we have established comprehensive lncRNA and circRNA expression atlases for *Sarcophaga peregrina* across various pupal stages. The inclusion of miRNA data in this study complements those efforts and further refines the non-coding RNA landscape of this species, providing a more complete molecular framework for advancing forensic entomology research.

In total, 191 known miRNAs were identified in this study. Differential expression analysis was conducted using two pairwise comparisons, early vs. middle pupal stages and middle vs. late pupal stages, which revealed 46 and 30 significantly differentially expressed miRNAs, respectively. Among these, nine miRNAs exhibited monotonic upregulation or downregulation across all three stages, suggesting potential as temporal markers. To further evaluate their correlation with pupal age, we collected additional developmental samples reared at a constant temperature of 25 °C and extracted miRNAs at 24 h intervals. Quantitative PCR validation identified six miRNAs—miR-92b, miR-210-3p, miR-275-5p, miR-285, miR-927-5p, and miR-956-3p—that showed strong temporal associations and stable expression trends, supporting their potential as reliable molecular markers for pupal age estimation.

In Dipteran flies, miRNAs such as miR-92b, miR-210-3p, miR-275-5p, miR-285, miR-927-5p, and miR-956-3p are involved in key physiological processes, including development, reproduction, immunity, and behavior. These miRNAs exhibit diverse functions with precise regulatory roles. In *Drosophila melanogaster* (Meigen, 1830) (Diptera: Drosophilidae), for instance, miR-133 overexpression induces hyperplasia of the larval wing disk by targeting genes such as phosphodiesterase 1C (PDE1C), thereby influencing cell proliferation and tissue growth pathways [19]. miR-285 is essential for nervous system development and homeostasis, particularly in maintaining blood–brain barrier (BBB) integrity, and is also implicated in photoperiod adaptation and circadian rhythm regulation [20,21,22]. miR-210, a highly conserved miRNA across taxa, is involved in metamorphic regulation, neural differentiation, and juvenile hormone signaling. Its upregulation has been linked to accelerated developmental progression [23,24,25,26]. miR-927 influences wing morphology and metamorphic timing in *Drosophila* by regulating downstream components of the juvenile hormone signaling pathway, such as *Krüppel-homolog* 1 (*Kr-h1*), as well as the Hippo pathway inhibitor Yorkie. It also plays important roles in immune responses and spermatogenesis [27,28,29,30,31]. miR-956 is highly expressed in intestinal progenitor cells of adult *Drosophila*. Loss-of-function studies have shown that disruption of miR-956 leads to an increased proportion of undifferentiated progenitor cells and a reduction in differentiated cell populations, indicating its role in maintaining the balance between stem cell renewal and differentiation, thereby preserving intestinal homeostasis [32,33]. miR-957 has been shown to play a crucial role in male courtship behavior in *Drosophila*. Male flies with miR-957 mutations exhibit markedly increased male–male courtship behaviors when housed with conspecific males, including persistent chain-like courtship behavior involving multiple individuals [34]. In addition, miR-957 is essential for female reproductive development. Loss of miR-957 function in female flies leads to impaired differentiation of germline stem cells and their progeny, resulting in the accumulation of undifferentiated germ cells within the ovarian germarium. These findings highlight the critical role of miR-957 in normal oogenesis and germ cell differentiation [35]. miR-92a regulates genes such as *shavenoid* and *jigr1*, thereby influencing wing and cuticular hair formation, neural stem cell maintenance, and memory consolidation in *Drosophila* [36,37,38,39,40,41,42]. In contrast, miR-92b forms a negative feedback loop with the transcription factor *Mef2*, which governs the developmental homeostasis of cardiac and somatic muscle tissues in the fly [37,43]. miR-275 is associated with metamorphosis and reproductive organ formation. miR-927 plays multifaceted roles in development, reproduction, and immunity. In *Drosophila*, its expression is inversely correlated with *Kr-h1*, suggesting it acts as a downstream effector of the juvenile hormone pathway to regulate growth and metamorphic transitions [28]. It also contributes to wing development, where overexpression in imaginal disks reduces wing size and induces apoptosis [30]. Functionally, miR-927 suppresses fecundity and increases adult mortality in *Drosophila* [28]. In *Zeugodacus cucurbitae* (Coquillett, 1899) (Diptera: Tephritidae), it is enriched in testes and its overexpression results in a ~50% reduction in sperm count [31]. Additionally, miR-927 is upregulated during chronic dengue infection in *Aedes albopictus* (Skuse, 1894) (Diptera: Culicidae), indicating a potential role in antiviral immune responses [27].

The differentially expressed miRNAs exhibited distinct temporal expression patterns throughout the pupal stage, likely reflecting stage-specific physiological events and underlying molecular regulatory mechanisms. Notably, miR-210-3p, miR-285, miR-927-5p, and miR-956-3p showed a continuous upregulation trend, peaking around days 9–10. This pattern suggests their potential involvement in key late-stage developmental processes such as tissue remodeling, neural maturation, metabolic reprogramming, and circadian rhythm coordination. For instance, the increased expression of miR-210 and miR-956 may be linked to the orchestration of metabolic transitions and circadian synchronization; the sustained elevation of miR-285 could facilitate glial proliferation and establishment of the blood–brain barrier; and the high expression of miR-927 may contribute to the termination of juvenile hormone signaling, thereby promoting the transition from pupa to adult. Collectively, these miRNAs demonstrate strong temporal specificity in mid-to-late pupal stages, highlighting their potential as molecular markers for age estimation during these critical developmental windows.

In contrast, miR-92b and miR-275-5p exhibited a marked decline in expression during the first 4–6 days of the pupal stage, subsequently stabilizing at low levels. Their elevated expression in early development suggests potential roles in initial organogenesis and maintenance of developmental homeostasis. The early high expression of miR-92b may be associated with the structural integrity of skeletal and cardiac muscle tissues, while miR-275-5p is likely involved in the establishment of gut barrier function and hormonal signaling responses. As organ systems progressively mature and attain functional stability, the expression of these miRNAs declines, indicating that their primary window of activity lies in the early to mid-pupal phases. Accordingly, miR-92b and miR-275-5p represent promising molecular markers for age estimation during the early-to-intermediate stages of pupal development.

This study offers valuable insights with direct relevance to forensic entomology. The six miRNAs identified herein exhibit well-defined and stage-specific temporal expression profiles, establishing them as promising molecular candidates for pupal age estimation. These results provide a molecular foundation for the development of miRNA-based age inference tools in necrophagous dipterans. The high coefficients of determination observed in our models likely reflect the tightly regulated and temporally specific expression of miRNAs during pupal development, rather than random variation or noise. Nevertheless, it is important to recognize potential limitations in the current modeling approach. The high R^2^ values (e.g., up to 0.997) observed in polynomial regression analyses may partially reflect overfitting due to the relatively small number of developmental time points and the use of fourth-order models. While these models perform exceptionally well under controlled laboratory conditions, their predictive accuracy and generalizability under real-world forensic scenarios remain to be validated. Future studies should incorporate cross-validation procedures, independent datasets, and potentially simpler modeling strategies to evaluate robustness and avoid inflating predictive confidence. In contrast to traditional morphological indicators, miRNAs confer significant advantages, including high tissue specificity and biochemical stability, which collectively enhance the accuracy, reproducibility, and resilience of age estimation methodologies under variable conditions [44]. Importantly, these miRNAs appear to function as intrinsic developmental timers, actively orchestrating key physiological transitions throughout metamorphosis. Their spatiotemporal robustness and relative resistance to environmental perturbations further highlight their translational potential in real-world forensic applications.

While this study provides a systematic characterization of the temporal expression dynamics of miRNAs during the pupal development of *Sarcophaga peregrina* and identifies promising molecular markers for age estimation, several limitations warrant consideration. Notably, all experiments were performed under constant laboratory conditions (25 °C), without accounting for critical environmental variables such as humidity, photoperiod, or diurnal temperature variation. This controlled setting may constrain the ecological validity and real-world applicability of the resulting predictive models. Accumulating evidence indicates that miRNA expression is modulated not only by genetic background but also by environmental cues [11]. Accordingly, future research should incorporate a broader range of both static and fluctuating environmental conditions to construct a context-resilient miRNA expression atlas. Such efforts would be instrumental in enhancing the generalizability, stability, and forensic utility of miRNA-based age estimation frameworks across diverse investigative scenarios.

Secondly, miRNA profiling in this study was performed using total RNA extracted from entire pupal bodies, without delineating expression variation across specific tissues or organs. However, growing evidence indicates that miRNAs often display pronounced spatial expression specificity, with distinct regulatory roles in tissues such as the nervous system, gut, and musculature [38]. This anatomical heterogeneity may mask key miRNA signals relevant to stage-specific physiological processes. Future investigations would benefit from integrating microdissection and tissue-resolved RNA extraction approaches to uncover anatomically localized miRNA signatures. Such refinement could substantially improve the resolution and biological interpretability of molecular models for pupal age estimation.

Moreover, our previous work has underscored the potential vulnerability of non-coding RNAs (ncRNAs) to postmortem degradation in pupal specimens [11]. In the present study, we observed notable fluctuations in ncRNA expression levels across dead pupae stored under varying conditions (e.g., −20 °C, −80 °C, or 4 °C), indicating that miRNAs may be subject to degradation during the death and preservation process. Yet, the kinetics of this degradation remain largely uncharacterized. Given that forensic samples are inherently derived from deceased individuals, the postmortem stability of miRNAs represents a pivotal determinant of their utility as temporal biomarkers. Future research should therefore pursue systematic characterization of miRNA stability under different decomposition stages and storage regimes to inform the development of standardized protocols for sample handling and robust forensic application.

In summary, future research should prioritize three critical avenues of inquiry: environmental adaptability, tissue-specific expression profiling, and postmortem molecular stability. Advancements in these domains will be instrumental in constructing a robust, generalizable, and field-deployable miRNA-based system for pupal age estimation. Such progress will significantly enhance the translational potential of miRNA biomarkers in forensic entomology, bridging the gap between molecular insights and real-world forensic applications.

## 5. Conclusions

In this study, we developed the first miRNA-based model for pupal age estimation in the necrophagous blowfly *Sarcophaga peregrina*. Through high-throughput sequencing and qRT-PCR validation, six miRNAs—miR-210-3p, miR-285, miR-927-5p, miR-956-3p, miR-92b, and miR-275-5p—were identified as tightly associated with pupal age, exhibiting consistent temporal expression patterns and strong model performance (R^2^ = 0.88–0.99). Functional enrichment analysis revealed that these miRNAs are involved in key regulatory pathways, including ecdysone signaling, metabolic reprogramming, and hypoxia response, underscoring their stage-specific regulatory roles during pupal development. Our findings demonstrate that miRNAs can serve as reliable temporal biomarkers for molecular age estimation in forensic entomology. Compared with conventional morphological approaches, miRNA-based indicators offer enhanced sensitivity and biochemical stability, presenting a novel and promising tool for postmortem interval (PMI) estimation. Moreover, this work lays a critical foundation for the future development of integrated, multimodal insect age estimation systems.

## Figures and Tables

**Table 1:** Primer sequences used for qRT-PCR validation of candidate miRNAs in *Sarcophaga peregrina*.

	Gene Name	Primer Sequences (5′→3′)	
Reference genes	U6	Forward	GGAACGATACAGAGAAGATTAGC
		Reverse	TGGAACGCTTCACGAATTTGCG
The DEmiRNAs *	miR-133	Forward	AGCTGGTTGACATCGGGTCA
	miR-285	Forward	TAGCACCATTCGAAATCAGTGC
	miR-92a	Forward	ATTGCACTTGTCCCGGCCTA
	miR-92b	Forward	AATTGCACTAGTCCCGGCCT
	miR-210-3p	Forward	TTGTGCGTGTGACAGCGG
	miR-275-5p	Forward	CGCGCTAATCAGTGACCGG
	miR-927-5p	Forward	GGCTTTAGAATTCCTACGCTTTACC
	miR-956-3p	Forward	GGCTTTAGAATTCCTACGCTTTACC
	miR-957-3p	Forward	TGAAACCGTCCAAAACTGAGGC

* miRNA qPCR 3′primer provided with miRNA 1st strand cDNA synthesis kit (AG11716, Accurate Biotechnology, Hunan, Co., Ltd., Changsha, China).

**Table 2:** Summary of fourth-order polynomial regression models for pupal-stage miRNA expression profiles.

Gene	Simulation Equation	F	*p*	R^2^
*miR-210-3p*	y = −2.14053 + 3.5806x − 0.35337x^2^ − 0.1x^3^ + 0.0133x^4^	35.81396	*p* < 0.001	0.96627
*miR-285*	y = 1.65554 − 1.59478x + 1.15842x^2^ − 0.23336x^3^ + 0.01419x^4^	46.49554	*p* < 0.001	0.97382
*miR-927-5p*	y = −3.81403 + 6.33601x − 1.64118x^2^ + 0.11845x^3^ + 0.00075x^4^	9.11545	*p* < 0.05	0.87941
*miR-956-3p*	y = −2.42511 + 4.23317x − 0.80865x^2^ − 0.00667x^3^ + 0.00725x^4^	13.90506	*p* < 0.01	0.91752
*miR-92b*	y = 0.64566 + 0.60861x − 0.29321x^2^ + 0.04075x^3^ − 0.0018x^4^	156.67715	*p* < 0.001	0.99208
*miR-275-5p*	y = 0.48393 + 0.83548x − 0.36654x^2^ + 0.04924x^3^ − 0.00212x^4^	410.45579	*p* < 0.001	0.99696

## Data Availability

Voucher specimens were deposited in the Forensic Entomology Laboratory of Yadong Guo’s research group at Central South University. Raw sequencing data have been submitted to the NCBI Sequence Read Archive (SRA) under the project accession number PRJNA820155. All scripts used for data processing, statistical modeling, and figure generation are available from the corresponding author upon reasonable request.

## Supplementary Materials

The following supporting information can be downloaded at: https://www.mdpi.com/article/10.3390/insects16080754/s1, Figure S1: Day 1 post-pupariation—White puparial stage. The puparium appears newly formed and pale white, with visible anterior spiracles. The cuticle is lightly sclerotized and fragile. Internal tissues are yellowish-white, and the head, thorax, and abdomen are indistinct. The puparium and internal body are tightly adhered, making dissection difficult. Figure S2: Day 2 post-pupariation—Yellow puparial stage. The puparium gradually turns yellow to brownish-yellow. Internal tissues remain tightly attached and fragile. The body inside is yellow-white; the head and thoracic structures remain undeveloped. The cephalopharyngeal skeleton is still tightly integrated with surrounding tissues. Figure S3: Day 3 post-pupariation—Cryptocephalic stage. The puparium can now be separated from the pupa more easily. The internal body is smooth and remains in a larval-like, shortened form. Legs and wing buds first appear as small protrusions. The head, thorax, and abdomen are still not clearly divided. Anterior spiracles become darker. Compound eyes are not yet visible. Figure S4: Day 4 post-pupariation—Phanerocephalic stage. The head, thorax, and abdomen begin to differentiate. Legs are thick, wing pads enlarge, occupying over half of the body length. Compound eyes become visible. The body appears white, and the puparial shell can be peeled off more easily without damage. Figure S5: Day 5 post-pupariation—Yellow-eye stage. Compound eyes begin to develop pigmentation, appearing light yellow. Antennal structure is more defined, and wing/leg structures are further elongated. The body surface remains smooth and hairless. Figure S6: Day 6 post-pupariation—Pink-eye stage. Compound eyes appear pink. Antennae are clearly contoured. The body is fully smooth with no visible bristles. Appendages are proportionally developed and lightly colored. Figure S7: Day 7 post-pupariation—Red-eye stage. Compound eyes deepen to red. Yellow bristles begin to emerge on the thorax and abdomen. Legs start to pigment. Antennae are fully developed and appear light yellow. Figure S8: Day 8 post-pupariation—Dark red-eye stage. The entire body takes on a yellowish hue. Thoracic and abdominal bristles are more prominent and appear light brown. Antennae and legs are light brown. Wings begin to pigment and show light gray coloration. Figure S9: Day 9 post-pupariation—Leg and wing darkening stage. Legs and wings show deep gray or gray-brown pigmentation. Bristles on the thorax and abdomen become dark brown to nearly black. Antennae deepen in color to brown. Compound eyes remain red. Figure S10: Day 10 post-pupariation—Brown-eye stage. Compound eyes turn brownish-red. The entire body darkens to gray-black. Antennae become dark brown to black. Legs and wings are fully black, and thoracic and abdominal bristles appear dark brown or black. Figure S11: Length distribution of small RNA sequencing reads; Figure S12: Predicted interactions between differentially expressed miRNAs and their target transcripts in *Sarcophaga peregrina* pupae; Table S1: Sequencing quality control (QC) summary; Table S2: Genome alignment statistics for small RNA sequencing data; Table S3: Summary of miRNA expression levels and annotations; Table S4: Classification statistics of known miRNAs; Table S5: TPM expression matrix of miRNAs across samples; Table S6: Predicted target genes of differentially expressed miRNAs (P3 vs. P1); Table S7: Predicted target genes of differentially expressed miRNAs (P5 vs. P3); Table S8: Summary of differentially expressed miRNAs and their predicted targets between developmental stages; Table S9: Expression trends of significantly upregulated miRNAs in Profile 11.
