# Supplementary material for: Temporal miRNA Biomarkers for Pupal Age Estimation in Sarcophaga peregrina (Diptera: Sarcophagidae)
Source: Insects. 2025 Jul 23;16(8):754. doi: 10.3390/insects16080754 (PMC12386374; doi:10.3390/insects16080754)
Supplement: Supplementary file 1 [file insects-16-00754-s001.zip › Supplementary Figure Legends.pdf]

Morphological Development of *Sarcophaga peregrina* During the Pupal Stage

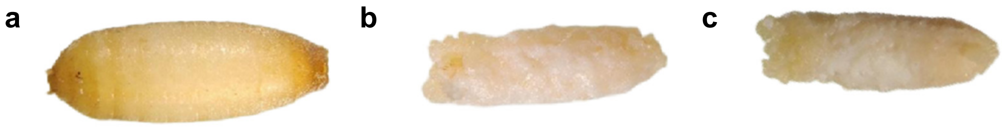

Supplementary Figure S1. Day 1 post-pupariation – White puparial stage
The puparium appears newly formed and pale white, with visible anterior spiracles. The cuticle is lightly sclerotized and fragile. Internal tissues are yellowish-white, and the head, thorax, and abdomen are indistinct. The puparium and internal body are tightly adhered, making dissection difficult.

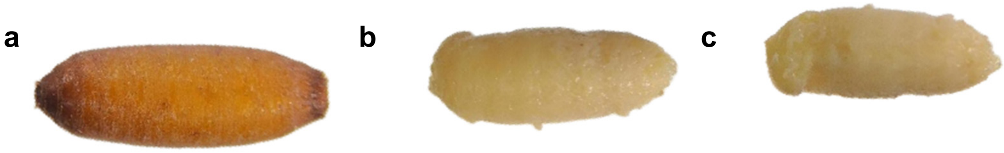

Supplementary Figure S2. Day 2 post-pupariation – Yellow puparial stage
The puparium gradually turns yellow to brownish-yellow. Internal tissues remain tightly attached and fragile. The body inside is yellow-white; the head and thoracic structures remain undeveloped. The cephalopharyngeal skeleton is still tightly integrated with surrounding tissues.

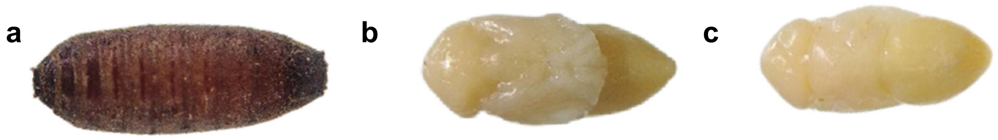

Supplementary Figure S3. Day 3 post-pupariation – Cryptocephalic stage
The puparium can now be separated from the pupa more easily. The internal body is smooth and remains in a larval-like, shortened form. Legs and wing buds first appear as small protrusions. The head, thorax, and abdomen are still not clearly divided. Anterior spiracles become darker. Compound eyes are not yet visible.

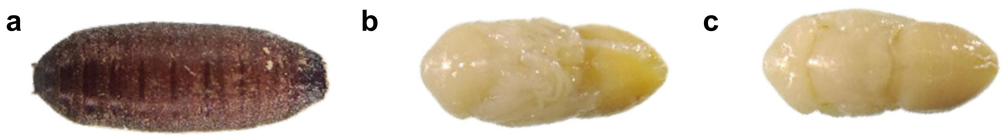

Supplementary Figure S4. Day 4 post-pupariation – Phanerocephalic stage
The head, thorax, and abdomen begin to differentiate. Legs are thick, wing pads enlarge, occupying over half of the body length. Compound eyes become visible. The body appears white, and the puparial shell can be peeled off more easily without damage.

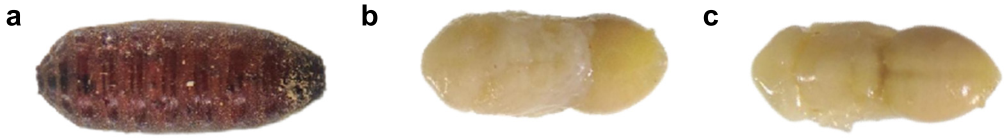

Supplementary Figure S5. Day 5 post-pupariation – Yellow-eye stage
Compound eyes begin to develop pigmentation, appearing light yellow. Antennal structure is more defined, and wing/leg structures are further elongated. The body surface remains smooth and hairless.

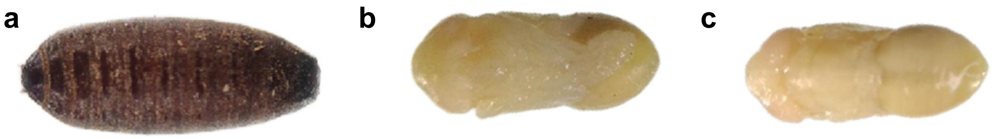

Supplementary Figure S6. Day 6 post-pupariation – Pink-eye stage
Compound eyes appear pink. Antennae are clearly contoured. The body is fully smooth with no visible bristles. Appendages are proportionally developed and lightly colored.

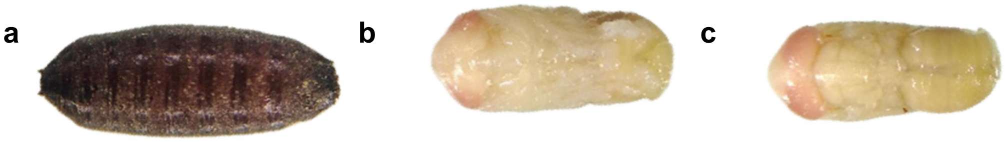

Supplementary Figure S7. Day 7 post-pupariation – Red-eye stage
Compound eyes deepen to red. Yellow bristles begin to emerge on the thorax and abdomen. Legs start to pigment. Antennae are fully developed and appear light yellow.

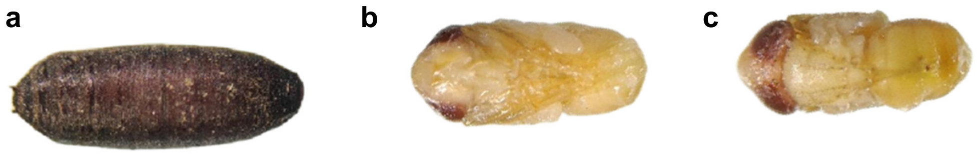

Supplementary Figure S8. Day 8 post-pupariation – Dark red-eye stage

The entire body takes on a yellowish hue. Thoracic and abdominal bristles are more prominent and appear light brown. Antennae and legs are light brown. Wings begin to pigment and show light gray coloration.

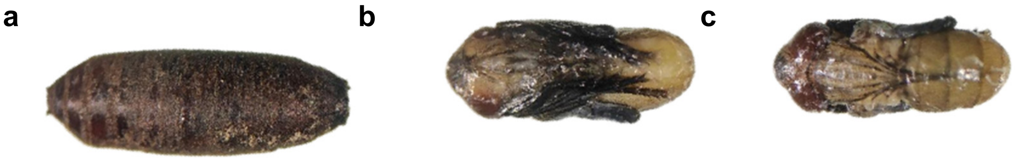

Supplementary Figure S9. Day 9 post-pupariation – Leg and wing darkening stage

Legs and wings show deep gray or gray-brown pigmentation. Bristles on the thorax and abdomen become dark brown to nearly black. Antennae deepen in color to brown. Compound eyes remain red.

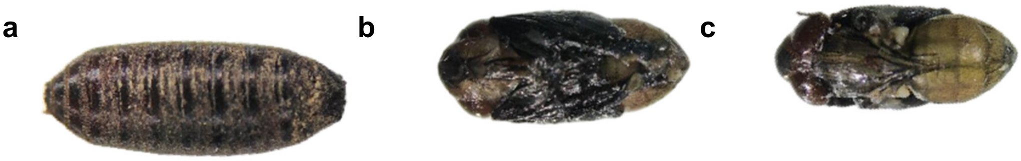

Supplementary Figure S10. Day 10 post-pupariation – Brown-eye stage

Compound eyes turn brownish-red. The entire body darkens to gray-black. Antennae become dark brown to black. Legs and wings are fully black, and thoracic and abdominal bristles appear dark brown or black.
