# Supplementary figures and images for: Temporal miRNA Biomarkers for Pupal Age Estimation in Sarcophaga peregrina (Diptera: Sarcophagidae)
Source: Insects. 2025 Jul 23;16(8):754. doi: 10.3390/insects16080754 (PMC12386374; doi:10.3390/insects16080754)

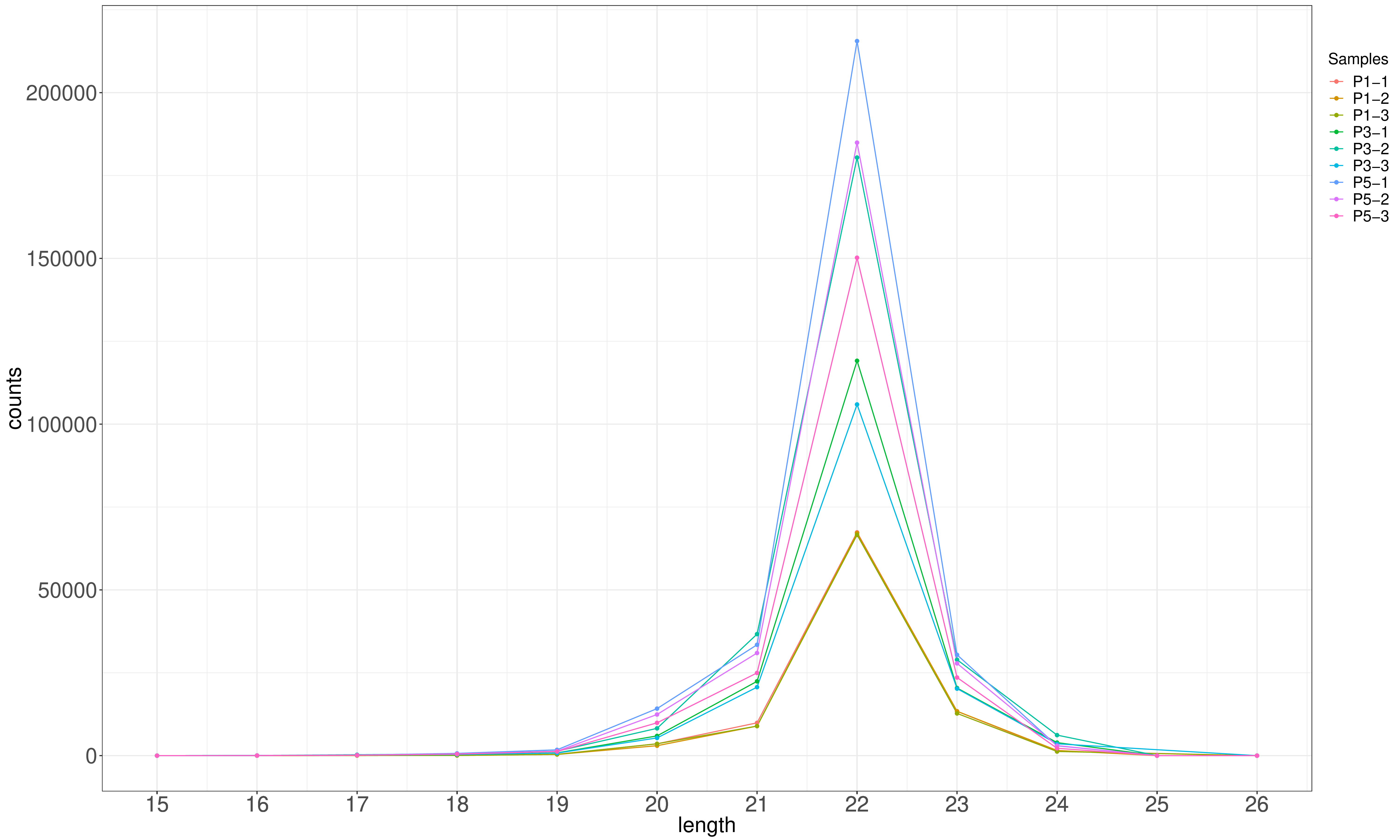

Supplement: Supplementary file 1 [file insects-16-00754-s001.zip › Figure S11. Length distribution of small RNA sequencing reads.png]

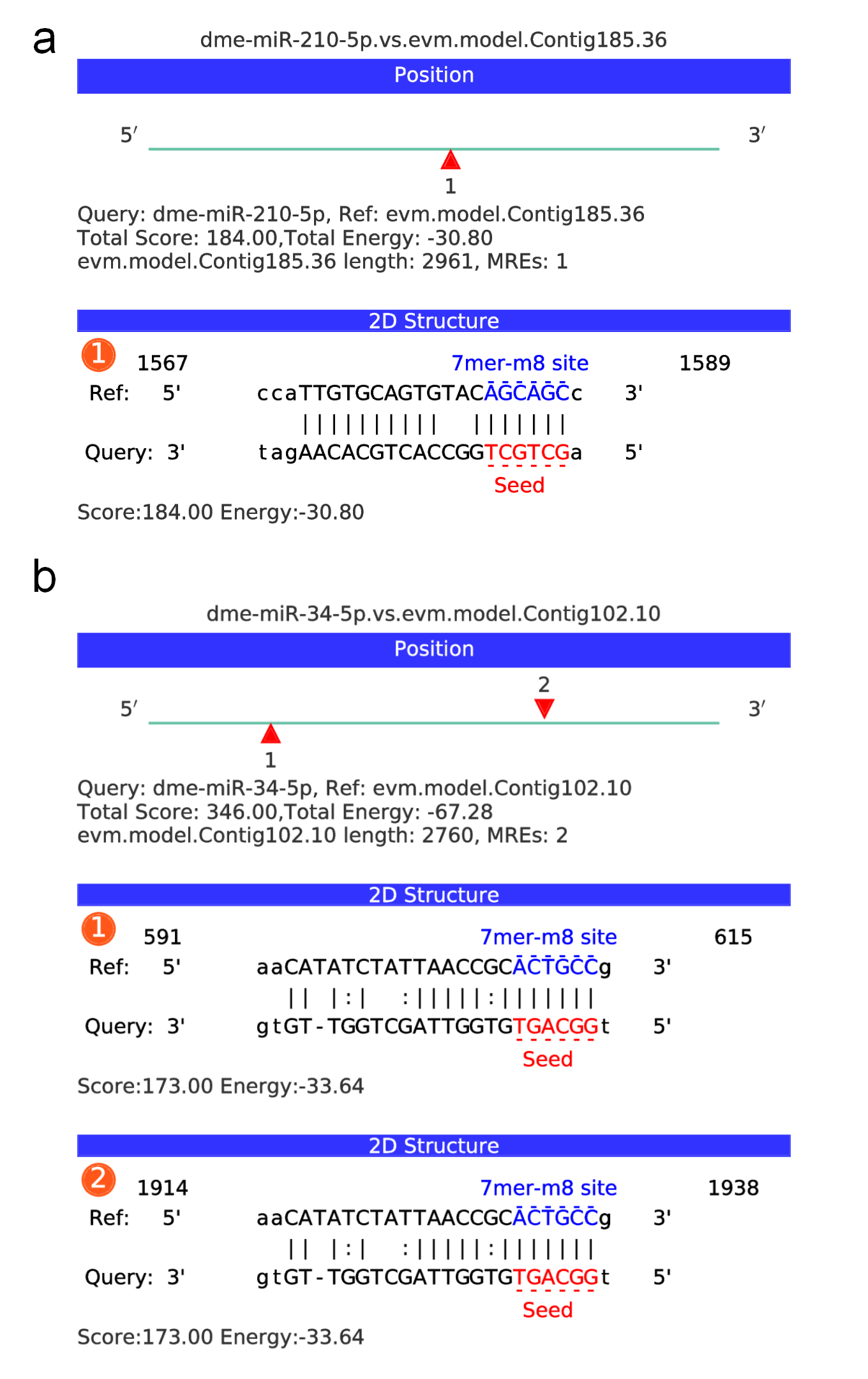

Supplement: Supplementary file 1 [file insects-16-00754-s001.zip › Figure S12. Predicted interactions between differentially expressed miRNAs and their target transcripts in Sarcophaga peregrina pupae.png]
